# Subtelomeric multiplex ligation-dependent probe amplification as a supplement for rapid prenatal detection of fetal chromosomal aberrations

**DOI:** 10.1186/s13039-014-0096-1

**Published:** 2014-12-09

**Authors:** Xiangnan Chen, Huanzheng Li, Yijian Mao, Xueqin Xu, Jiaojiao Lv, Lili Zhou, Xiaoling Lin, Shaohua Tang

**Affiliations:** 1 School of Laboratory Medicine and Life Science, Wenzhou Medical University, Key Laboratory of Medical Genetics, Zhejiang, China; 2 Department of Genetics, Dingli Clinical Medical School, Wenzhou Medical University, Key Laboratory of Birth Defects, Wenzhou, Zhejiang 325000 China

**Keywords:** Subtelomeric MLPA, Fetal chromosomal aberrations, Aneuploidy, Rearrangements, Mosaics, sSMC, High-risk fetuses, SNP array

## Abstract

**Background:**

Pregnant women with high-risk indications are highly suspected of fetal chromosomal aberrations. To determine whether Multiplex Ligation-dependent Probe Amplification (MLPA) using subtelomeric probe mixes (P036-E2 and P070-B2) is a reliable method for rapid detection of fetal chromosomal aberrations. The subtelomeric MLPA probe mixes were used to evaluate 50 blood samples from healthy individuals. 168 amniocytes and 182 umbilical cord blood samples from high-risk fetuses were analyzed using the same subtelomeric MLPA probe sets. Karyotyping was also performed in all cases of high-risk pregnancies, and single nucleotide polymorphism array analysis was used to confirm submicroscopic and ambiguous results from MLPA/karyotyping.

**Results:**

Subtelomeric MLPA analysis of normal samples showed normal result in all cases by use of P036-E2 probe mix, while P070-B2 probe mix gave normal results for all but one case. In one normal control case P070-B2 produced a duplicated signal of probe for 13q34. In the high-risk group, totally 44 chromosomal abnormalities were found by karyotyping and MLPA, including 23 aneuploidies and 21 rearrangements or mosaics. MLPA detected all 23 aneuploidies, 12 rearrangements and 1 mosaic. Importantly, MLPA revealed 4 chromosomal translocations, 2 small supernumerary marker chromosomes (sSMCs), and 3 subtelomeric imbalances that were not well characterized or not detectable by karyotyping. However, MLPA showed negetive results for the remaining 8 rearrangements or mosaics, including 3 low mosaic aneuploidies, 1 inherited sSMC, and 4 paracentric inversions.

**Conclusions:**

Results suggest that combined use of subtelomeric MLPA and karyotyping may be an alternative method for using karyotype analyses alone in rapid detection of aneuploidies, rearrangements, and sSMCs.

**Electronic supplementary material:**

The online version of this article (doi:10.1186/s13039-014-0096-1) contains supplementary material, which is available to authorized users.

## Background

Pregnant women greater than 35 years old have increased risk of aneuploid pregnancies, particularly trisomy 21, which produces Down’s syndrome, and trisomy 18. Positive screening of serological markers (AFP and β-hCG), history of affected children, parental chromosomal rearrangement, and ultrasound identified anomalies are used to test for fetal chromosomal aberrations in high-risk pregnancies [1]. In China, amniocytes collected by amniocentesis at 14–23 weeks gestation or umbilical cord blood samples obtained by cordocentesis at 24–28 weeks are used for cytogenetic analyses to detect fetal chromosomal abnormalities [2].

Karyotyping cultured amniocytes has been the gold standard for studying fetal chromosomal aberrations since the 1970s [3]. However, the technique presents some limitations, such as low resolution (>5 Mb), time-consumption (10–14 weeks), and culture failure (0.2-0.6%) [4]. Over the last two decades, probe-based tests, including quantitative fluorescence polymerase chain reaction (QF-PCR) and fluorescence *in situ* hybridization (FISH), have been used to supplement karyotyping for rapid detection of aneuploidies [5,6]. Compared to QF-PCR and FISH, Multiplex Ligation-dependent Probe Amplification (MLPA) is high-throughput, and enables analysis of more than 40 genomic locations simultaneously using only small (20 ng) amounts of DNA [7]. MLPA is also able to distinguish sequences differing in only a single nucleotide and detect small copy number differences, rapid turnaround time which means results can be obtained within 2 work days [8,9]. Two studies using MLPA detects big cohort of prenatal samples (3925 and 4000, respectively )with high risk indications showed that MLPA is a reliable method that can replace FISH and karyotyping used as large scale testing for rapid aneuploidy diagnosis [10,11]. Other studies shows that addition MLPA probe mixes targeted subtelomeric regions and microdeletion syndromes can increase detection of pathogenic rearrangements which are valuable in routine prenatal diagnostics [12-14].

Subtelomeric MLPA probe mixes contain probes for subtelomeric or pericentromeric regions, which were designed to detect subtelomeric rearrangements. Postnatally, the probe mixes are already used in clinical genetics for detection of copy number variations (CNVs) in subtelomeric regions, which are gene-rich and prone to rearrangement in patients with intellectual disability [15-17]. For prenatal diagnostics, detection of DNA from prenatal samples also showed the probe sets have potential to detect common aneuploidies and increase detection of chromosomal rearrangements [2,13]. However, the presence of CNVs in the human genome has been widely demonstrated, and different ethnic populations may present different polymorphisms [18,19]. To determine whether subtelomeric MLPA is suitable for the Chinese population, P036-E2 and P070-B2 probe sets were evaluated. In this study,we used subtelomeric MLPA as a supplementary method to karyotyping for the detection of fetal chromosomal aberrations in second-trimester high-risk pregnancies.

## Results

### Subtelomeric MLPA probe set evaluation

In total 50 DNA samples extracted from peripheral blood of healthy individuals were analyzed. P036-E2 MLPA subtelomeric probe mix showed normal results for all analyzed samples. In one case P070-B2 MLPA subtelomeric probe mix showed duplication of the probe located in 13q34 (CDC16-8). The relative ratio of each probe signal was 1 ± 0.01 SD <0.1 (Additional file 1).

### High-risk pregnancy samples

In the high-risk pregnancy group, 44 (12.6%) of 350 cases were found to have chromosomal aberrations using the combination of MLPA probe sets and karyotyping. Detected abnormalities included 23 aneuploidies and 21 rearrangements or mosaics (Table 1 and Table 2).

**Table 1:** **Karyotyping and subtelomeric MLPA results for the 23 fetuses with aneuploidy**

**Karyotype**	**Cases**	MLPA results*	
		**P036-E2**	**P070-B2**
Trisomy 21	12	Dup 21p(RBM11-1)	Dup 21q(HSPA13-2)
		Dup 21q(PRMT2-4)	Dup 21q(S100B-2)
Trisomy 18	4	Dup 18p(USP14-7)	Dup 18p(SECTM1-21)
		Dup 18q(RBFA-4)	Dup 18q(THOC1-9)
Trisomy 13	1	Dup 13q(PSPC1-2)	Dup 13q(PSPC1-1)
		Dup 13q(F7-6)	Dup 13q(CDC16-8)
Monosomy X	2	Del X/Yp(SHOX-4)	Del X/Yp(SHOX-5)
		Del X/Yq(VAMP7-4)	Del X/Yq(VAMP7-8)
47,XXX	1	Dup X/Yp(SHOX-4)	Dup X/Yp(SHOX-5)
		Dup X/Yq(VAMP7-4)	Dup X/Yq(VAMP7-8)
47,XXY	2	Dup X/Yp(SHOX-4)	Dup X/Yp(SHOX-5)
		Dup X/Yq(VAMP7-4)	Dup X/Yq(VAMP7-8)
		One copy of Yp(ZFY-4)	One copy of Yq(DDX3Y-18)
47,XYY	1	Dup X/Yp(SHOX-4)	Dup X/Yp(SHOX-5)
		Dup X/Yq(VAMP7-4)	Dup X/Yq(VAMP7-8)
		Dup Yp(ZFY-4)	Dup Yq(DDX3Y-18)

*Results of probe variations were described with format as: variation type, chromosomal arm and gene-exon (within parentheses).Dup: duplicated. Del: deleted.

**Table 2:** **Details of the 21 rearrangements or mosaics detected by full karyotyping and subtelomeric MLPA**

**Karyotype on cultured cells**	**Sample type** ^**^	**Indication** ^***^	**MLPA results**	
			**P036-E2**	**P070-B2**
46,XX	Amnio	UA	Del 7q(VIPR2-3)	Del 7q(VIPR2-2)
46,XY	Amnio	UA	Dup 16p(POLR3K-1)	Dup 16p(DECR2-9)
46,XX	CB	UA	Del 11q(NCAPD3-2)	Del 11q(IGSF9B-20)
			Dup 9q(EHMT1-24)	Dup 9q(EHMT1-10)
45,XX,psu dic(4;22)(p11;p11.2)	CB	UA	Del 4p(PIGG-7)	Del 4p(PIGG-8)
46,XX,del (5)(p13)	CB	UA	Del 5p(PDCD6-6)	Del 5p(CCDC127-3)
46,XX,del(10)(q26)	Amnio	PHGI	Del 10q(PAOX-3)	Del 10q(ECHS1-8)
46,XX,del(18)(p10)	Amnio	IASR	Del 18p(USP14-7)	Del 18p(THOC1-21)
46,XY,add(2) (q37)	Amnio	UA	Del 2q(CAPN10-3)	Del 2q(ATG4B-7)
			Dup 3p(CHL1-5)	Dup 3p(CHL1-3)
46,XX,add(14) (q32)	Amnio	PTC	Dup 4q(TRIML2-2)	Dup 4q(FRG1-1)
			Del 14q(MTA1-8)	Del 14q(MTA1-7)
46,XY,add(21) (q22)	CB	IASR	Dup 17q(TBCD-18)	Dup 17q(SECTM1-4)
			Del 21q(PRMT2-4)	Del 21q(S100B-2)
46,XX,add(6)(q27)	Amnio	PHGI	Del 6q(PSMB1-5)	Del 6q(TBP-2)
			Dup 18p(USP14-7)	Dup 18p(THOC1-21)
mos 47,XY,+mar[9]/46,XY[12]	Amnio	IASR’	Dup 21q(RBM11-1)	Dup 21q(HSPA13-2)
46,X,+mar	Amnio	IASR	Dup X/Yp(SHOX-4)	Dup X/Yp(SHOX-5)
			Del X/Yq(VAMP7-4)	Del X/Yq(VAMP7-8)
			Dup Yp(ZFY-4)	Dup Yq(DDX3Y-18)
46,XN,inv(9)^*^	Amnio	IASR	Normal	Normal
46,XX,inv(7)(q22q31)(mat)	Amnio	PHGI	Normal	Normal
47,XX,+mar(pat)	Amnio	IASR	Normal	Normal
mos 47,XX,+21[5]/46,XX[15]	Amnio	IASR	Normal	Normal
mos 47,XX,+7[3]/46,XX,[17]	Amnio	IASR	Normal	Normal
mos 45,X[6]/46,XY[17]	Amnio	HRA	Normal	Normal

*3 femal and 1 male fetuses with 46,XN,inv(9).

**Amnio: amniocyte; CB: cord blood.

***UA: Ultrasound abnormality; PTC: Parental translocation carrier; HRA: High-risk age; IASR: Increased aneuploidy screening risk; PHGI: Past history of genetic indications.

mos: mosaic; mar: marker.

#### Karyotyping results

Karyotyping alone detected 41 chromosomal abnormalities, including 23 aneuploidies, 8 terminal imbalanced rearranggements, 4 mosaics, 4 paracentric inversions and 3 sSMCs(one was mosaic). Different chromosomal band was involved in the 8 terminal imbalanced rearranggements, including 2q, 4p, 5p, 6q, 10q, 14q, 18p and 21q. Level of the 4 mosaics are 15%, 25%, 26% and 42%, respectively. In the 4 paracentric inversions, 3 involved chromosome 9 and the remaining one involved chromosome 7 which was inherited from a healthy mother.

#### Subtelomeric MLPA anlaysis

**Figure 1:**
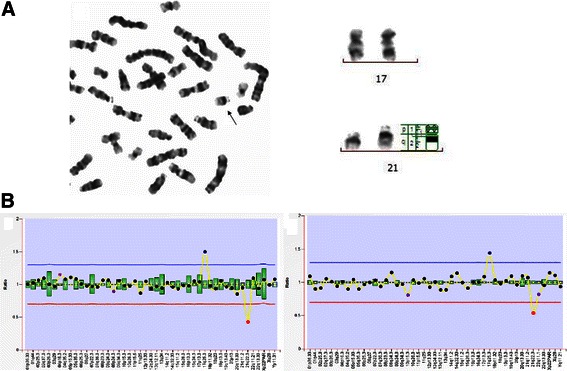
**Identification of chromosomal rearrangements with karyotype analyses and MLPA. (A)** Karyotype analysis showed a derived chromosome 21 (black arrow). Band analyses revealed the derived chromosome was longer than chromosome 21 and the two copies of chromosome 17 were normal. **(B)** MLPA results with probe P036-E2 (left) and P070-B2 (right). Probes targeting 17q25.3 (blue dot) were increased and the signals for 21q22.3 (red dot) were decreased, indicating unbalanced translocation with deletion of 21qter and duplication of 17qter.

MLPA detected all the 23 aneuploidies and each showed at least two probe changes for one MLPA probe set (Table 1). And 12 rearrangements and 1 mosaic were also detected, 3 of which were missed by karyotype analyses and 10 of which were both detected by MLPA and karyotyping (Table 2). MLPA revealed unbalanced chromosomal translocations in 4 of the rearrangements that were not microscopically detectable: 2qter deletion and 3pter duplication (46,XY,add(2)(q37)); 14qter deletion and 4qter duplication (46,XX,add(14)(q32)); 21qter deletion and 17qter duplication (46,XY,add(21)(q22)) (Figure 1); 6qter deletion and 18pter duplication (46,XX,add(6)(q27)). Two of the sSMCs, one of which was mosaic(42%), were also identified by MLPA. However, MLPA showed negative results for three low mosaic aneuploidies (15%, 25%, and 26%), one inherited sSMC and all 4 paracentric inversions, which were detected by karyotyping.

#### SNP array analyses

SNP array analyses were performed for the 3 rearrangements that were revealed by MLPA but not detected by karyotyping. Results confirmed the deletion of 7qter with a minimum size of 0.1 Mb, Chr7:g. 158064729–159119486; duplication of 16pter with a minimum size of 0.3 Mb, Chr16:g. 152220–480875; and partial monosomy of 11q and partial trisomy of 9q. SNP array analyses also revealed that the 11q deletion spanned approximately 10.2 Mb, and the 9q duplication spanned 12.9 Mb.

## Discussion and conclusions

To improve detection rates of chromosomal aberrations for high-risk pregnancies, new rapid and reliable prenatal diagnostic tests are needed. Many different diagnostic approaches have been reported, including QF-PCR, targeted FISH, and genome wide technologies such as SNP arrays and comparative genomic hybridization [20-22]. This study focused on MLPA as a supplement for current tests. This technology is high-throughput, with up to 96 samples analyzed in each run [23]. It is also economical, easy to perform, reproducible, and sensitive.

Totally 400 DNA samples (50 blood samples from healthy individuals and 350 prenatal samples) were assayed. The 50 blood samples were assayed in a single experiment, and the results, including data analyses, were available in 48 hours. We found one case showed duplication of the probe located in 13q34 (CDC16-8) for P070-B2 MLPA subtelomeric probe mix. Based on the database of genomic variants (DGV:dgv.tcag.ca/gb2/gbrowse/dgv2_hg19/), this duplicated region may be a benign copy number variation in normal individual. However, variability in probe signals can occur due to variations in sample purity and experimental conditions. There is no further validation has been made, this may be a false positive results detected by MLPA.

Results of subtelomeric MLPA analysis of 350 DNA samples from high-risk pregnancies were analyzed by subtelomeric MLPA, revealing 36 abnormal results, including 23 aneuploidies and 13 rearrangements or mosaics. These findings demonstrate that subtelomeric MLPA can identify all major aneuploidies and many chromosomal rearrangements that are not well characterized by karyotyping or not detectible by karyotyping.

MLPA is especially useful for characterizing unbalanced rearrangements as a complement to conventional cytogenetics [12]. Indeed, MLPA detected unbalanced subtelomeric translocations in 4 cases of deletion and duplication, but karyotyping only showed some addition in one of the chromosomes. Characterization of this type of rearrangement with short turnaround time is crucial for prenatal diagnoses.

Detection of mosaics by MLPA is dependent on the level of the mosaic. In this study, four mosaics detected by karyotyping had levels of 42%, 25%, 15%, and 26%, and only the 42% mosaic sSMC was detected by subtelomeric MLPA. It is consistent with previous study that MLPA can detect duplicated mosaics to a level as low as 40% [24].

sSMC detection was also tested. Three sSMCs were detected by karyotyping, and 2 of them were also detected by subtelomeric MLPA. Parental karyotyping revealed that the 2 positive sSMCs were *de novo*. Previous studies showed that both centromeric and subtelomeric MLPA probe sets can rapidly distinguish unique-sequence positive and negative sSMCs [25]. However, Detection of aberration by MLPA technique is limited by probe location.

Cryptic, unbalanced subtelomeric rearrangements have been identified as an important contributor (0.5-4.1%) to the etiology of fetal malformations [8,26,27]. In this study, 3 (0.8%) normal karyotypes were found to have chromosomal imbalances by MLPA, including a 7qter deletion, a 16pter duplication, and an unbalanced translocation with 11qter deletion and 9qter duplication. Further characterization of the 7qter deletion and 16pter duplication using SNP arrays revealed that these two imbalances were smaller than 3 Mb, what is usually under detection resolution of conventionally by karyotyping. Partial monosomy 11q and partial trisomy 9q should be detectable using high-resolution karyotype analysis, because SNP arrays revealed that they spanned approximately 10.2 Mb and 12.9 Mb, respectively. Using this information, it was deduced that the derivative chromosome was 2.7 Mb longer than chromosome 11. The replacement region, 9q33.3q34.3, stained similar to region 11q24.2q25 in cytogenetic analyses at a resolution of 350–400 bands. In this particular case it is possible that this led to a misdiagnosis by karyotype analysis.

Using subtelomeric MLPA probe mix in prenatal diagnoses allows a rapid fetal chromosomal analysis and possible it could increase detection rates for chromosomal rearrangements. However, the precise breakpoints of the rearrangements can’t be established by either karyotyping or MLPA. SNP array analysis may overcome this obstacle.

SNP array testing is faster and allows detection of much smaller aberrations (approximately 0.15 Mb) compared to karyotyping (approximately >5 Mb) [28,29]. However, SNP technology is costly and technically complex. Therefore, it is not considered suitable for routine diagnostic use in developing countries. In contrast, subtelomeric MLPA, as a supplementary tool for karyotype analyses to detect fetal chromosomal aberrations, is feasible in diverse settings. In addition, MLPA probemix P290 Prenatal Microdeletions has been developed to simultaneously screen prenatal samples for trisomies 13, 18, 21 and for several microdeletion syndromes. Combined using this probemix with subtelomeric MLPA probemixes may be more reliable for aneuploidy detection and increase diagnostic yield for chromomal rearrangements. However, for confirming chromosomal rearrangements detected by karyotyping/MLPA, or analyzing suspected aberrations not revealed by MLPA/karyotyping, genome-wide array methods may be a good choice.

## Methods

### Materials

Fifty peripheral blood samples were collected from healthy individuals (39 males, 11 females, average age is 27, range: 23 to 35) at the Health Check Centre of Wenzhou Central Hospital. In total 350 pregnant women underwent fetal karyotype analyses by amniocentesis or cordocentesis. The average age of pregnant women was 28, and the range was 20 to 35. 168 samples of amniotic fluid were obtained at an average gestation of 20 weeks (range: 17 to 23). 182 cord blood samples were obtained at 24 to 30 weeks gestation (average: 26 weeks). Patients were classified into five groups based on prenatal indications (Table 3). All patients were informed about the study, and written consents for participation were obtained. This study is approved by the Dingli Clinical School Ethics Committee of Wenzhou Medical University (NO.201410).

**Table 3:** **Classification of the high-risk pregnancy samples according to prenatal indications**

**Classifications**	**Amniocytes**	**Umbilical cord blood**
Ultrasound abnormality	54	90
Increased aneuploidy screening risk	62	31
High age risk	32	28
Past history of genetic indications	17	32
Parental translocation carrier	3	1
Total	168	182

### Karyotype analyses

All prenatal samples were cultured following standard protocols [3]. Amniocytes were cultured with BIOAMF-2 medium (Biological Industry, Kibbutz Beit Haemek, Israel), and cord blood cells were cultivated with peripheral blood lymphocyte medium (Xiangya gene technology, Hunan, China). At least 20 G-banded metaphases from each sample were analyzed using G-banding Wright-staining method. Karyotypes were analyzed by two senior laboratory technicians.

### DNA for MLPA

Five ml uncultured amniotic fluid or 200 μl blood samples were used to isolate DNA with the QIAamp DNA Mini kit (Qiagen, Germany). Purified DNA was eluted with 50–200 μl of AE buffer. Sample concentration and purity were detected using a NanoDrop 2000UV-vis spectrophotometer (Thermo Scientific, USA).

### MLPA

Subtelomeric MLPA probe sets, P036-E2 and P070-B2, were supplied by MRC-Holland (http://www.mlpa.com). Each probe set contains one MLPA probe for subtelomeric or pericentromeric regions, which are primarily designed to detect deletions/duplications. We used at least 2 reference samples in each MLPA run. When using more than 8 samples, added 1 additional reference sample for each 3 samples. Reference samples are DNA from nomal controls. DNA was diluted to 20–30 ng/μl and 5 μl DNA was used in each MLPA reaction. An ABI-2720 thermocycler (USA) was used for MLPA, following the manufacturer’s instructions. ABI 3130 was used for fragment separation and the parameters were set as follows: Injection voltage: 1.6 kV; injection time: 15 seconds; run voltage: 15 kV; polymer: POP7; run voltage: 10 kV, and run time: 2000 seconds. Data was analyzed using Coffalyser.NET software developed by the manufacturer. All analytical parameters were set to default. The algorithm of Coffalyser.NET primarily runs two steps: firstly, data for each test probe of each sample will be compared to each available reference sample, producing as many dosage quotients as there are reference samples;secondly, final ratio will then estimated by calculating the average over these dosage quotients. In this study, arbitrary ratio border (low: 0.70; high: 1.30) was used for the final ratio to determine whether an abnormal result was detected.

### Single nucleotide polymorphism (SNP) array

We used the HumanCytoSNP-12 v1.0 (Illumina, USA) SNP array platform, which contains 298,563 tagSNP markers with an average resolution of 31 Kb. Two hundred ng DNA was used as input for each array. DNA amplification, tagging, and hybridization were performed according to manufacturer’s protocols. Array slides were scanned on an iScan reader. Data analysis was performed using GenomeStudio (version 2010.1). The breakpoint positions of each aberrant region were converted to Genome Reference Consortium Human Build 37 patch release 13 (GRCh37.p13).

## Additional file

Additional file 1:
**Data of each probe collected from healthy individuals by performing subtelomeric MLPA.**
