# Erratum to: Subtelomeric multiplex ligation-dependent probe amplification as a supplement for rapid prenatal detection of fetal chromosomal aberrations

**DOI:** 10.1186/s13039-016-0290-4

**Published:** 2016-11-14

**Authors:** Xiangnan Chen, Huanzheng Li, Yijian Mao, Xueqin Xu, Jiaojiao Lv, Lili Zhou, Xiaoling Lin, Shaohua Tang

**Affiliations:** 1 School of Laboratory Medicine and Life Science, Wenzhou Medical University, Key Laboratory of Medical Genetics, Zhejiang, China; 2 Department of Genetics, Dingli Clinical Medical School, Wenzhou Medical University, Key Laboratory of Birth Defects, Wenzhou, Zhejiang 325000 China

## Erratum

Unfortunately, the original version of this article [1] contained an error. The Acknowledgement section contained the incorrect funding number for the Ministry of Health Project, instead of NO.WKJ2011-2-017 it should be NO.WKJ2011-2-019. The corrected Acknowledgment section can be found below.
