# Supplementary material for: Subtelomeric multiplex ligation-dependent probe amplification as a supplement for rapid prenatal detection of fetal chromosomal aberrations
Source: Mol Cytogenet. 2014 Dec 9;7:96. doi: 10.1186/s13039-014-0096-1 (PMC4265491; doi:10.1186/s13039-014-0096-1)
Supplement: Additional file 1: — Data of each probe collected from healthy individuals by performing subtelomeric MLPA. [file 13039_2014_96_MOESM1_ESM.docx]

Additional file 1: Data of each probe collected from healthy individuals by performing subtelomeric MLPA.

| Probes P036-E2 | | Relative ratio* (N = 50) | | SD | Probes P070-B2 | | Relative ratio* (N = 50) | | SD |
| --- | --- | --- | --- | --- | --- | --- | --- | --- | --- |
| Gene | Chr.band | Minimum | Maximum |  | Gene | Chr.band | Minimum | Maximum |  |
| ZFY**  TNFRSF4  SH3BP5L  ACP1  CAPN10  CHL1  BDH1  PIGG  TRIML2  PDCD6  GNB2L1  IRF4  PSMB1  ADAP1  VIPR2  FBXO25  ZC3H3  DMRT1  EHMT1  DIP2C  PAOX  RIC8A  NCAPD3  SLC6A12  ZNF10  PSPC1  F7  CCNB1IP1  MTA1  MKRN3  ALDH1A3  POLR3K  GAS8  RPH3AL  TBCD  USP14  RBFA  CDC34  CHMP2A  SOX12  OPRL1  RBM11  PRMT2  BID  RABL2B  SHOX  VAMP7 | Yp11.31  01p36.33  01q44  02p25.3  02q37.3  03p26.3  03q29  04p16.3  04q35.2  05p15.33  05q35.3  06p25.3  06q27  07p22.3  07q36.3  08p23.3  08q24.3  09p24.3  09q34.3  10p15.3  10q26.3  11p15.5  11q25  12p13.33  12q24.33  13q12.11  13q34  14q11.2  14q32.33  15q11.2  15q26.3  16p13.3  16q24.3  17p13.3  17q25.3  18p11.32  18q23  19p13.3  19q13.43  20p13  20q13.33  21q11.2  21q22.3  22q11.21  22q13.33  Xp22PAR  Xq28 | 0.99  0.83  0.94  0.89  0.9  0.91  0.91  0.94  0.86  0.96  0.96  0.92  0.93  0.78  0.91  0.91  0.96  0.93  0.93  0.92  0.88  0.93  0.92  0.97  0.91  0.93  0.94  0.89  0.87  0.87  0.9  0.92  0.94  0.95  0.93  0.92  0.93  0.88  0.88  0.89  0.88  0.85  0.88  0.93  0.88  0.91  0.88 | 1.18  1.08  1.07  1.2  1.06  1.17  1.13  1.12  1.26  1.09  1.15  1.06  1.15  1.15  1.13  1.14  1.11  1.05  1.07  1.06  1.09  1.06  1.08  1.17  1.07  1.06  1.09  1.09  1.11  1.07  1.15  1.07  1.12  1.08  1.13  1.08  1.07  1.21  1.12  1.2  1.09  1.15  1.07  1.08  1.13  1.11  1.08 | 0.09  0.05  0.03  0.07  0.04  0.07  0.05  0.03  0.1  0.03  0.05  0.04  0.06  0.07  0.05  0.05  0.04  0.03  0.03  0.03  0.04  0.03  0.04  0.04  0.04  0.03  0.04  0.05  0.05  0.04  0.06  0.04  0.04  0.03  0.04  0.03  0.03  0.07  0.07  0.08  0.05  0.06  0.04  0.04  0.07  0.05  0.05 | DDX3Y**  TNFRSF18  SH3BP5L  ACP1  ATG4B  CHL1  KIAA0226  PIGG  FRG1  CCDC127  GNB2L1  IRF4  TBP  SUN1  VIPR2  FBXO25  RECQL4  DOCK8  EHMT1  ZMYND11  ECHS1  BET1L  IGSF9B  KDM5A  ZNF10  PSPC1  CDC16  PARP2  MTA1  NDN  TM2D3  DECR2  GAS8  RPH3AL  SECTM1  THOC1  CTDP1  PPAP2C  CHMP2A  ZCCHC3  UCKL1  HSPA13  S100B  IL17RA  ARSA  SHOX  VAMP7 | Yq11.21  01p36.33  01q44  02p25.3  02q37.3  03p26.3  03q29  04p16.3  04q35.2  05p15.33  05q35.3  06p25.3  06q27  07p22.3  07q36.3  08p23.3  08q24.3  09p24.3  09q34.3  10p15.3  10q26.3  11p15.5  11q25  12p13.33  12q24.33  13q12.11  13q34  14q11.2  14q32.33  15q11.2  15q26.3  16p13.3  16q24.3  17p13.3  17q25.3  18p11.32  18q23  19p13.3  19q13.43  20p13  20q13.33  21q11.2  21q22.3  22q11.1  22q13.33  Xp22PAR  Xq28 | 0.84  0.86  0.93  0.91  0.95  0.94  0.93  0.91  0.95  0.92  0.95  0.93  0.93  0.93  0.95  0.95  0.84  0.89  0.94  0.88  0.95  0.9  0.92  0.93  0.93  0.86  0.89  0.93  0.94  0.94  0.87  0.79  0.95  0.93  0.93  0.95  0.82  0.93  0.9  0.83  0.94  0.89  0.93  0.93  0.91  0.89  0.89 | 1.14  1.12  1.05  1.13  1.05  1.12  1.15  1.08  1.19  1.08  1.04  1.17  1.06  1.11  1.06  1.07  1.1  1.07  1.13  1.11  1.05  1.06  1.03  1.12  1.07  1.14  **1.52*****  1.09  1.08  1.06  1.09  1.07  1.04  1.06  1.09  1.1  1.11  1.11  1.1  1.16  1.07  1.13  1.06  1.18  1.17  1.07  1.13 | 0.08  0.05  0.03  0.05  0.03  0.05  0.05  0.04  0.06  0.04  0.02  0.05  0.03  0.04  0.03  0.03  0.05  0.04  0.05  0.06  0.02  0.04  0.03  0.04  0.03  0.07  0.11  0.04  0.04  0.03  0.05  0.06  0.02  0.03  0.04  0.04  0.05  0.03  0.05  0.08  0.04  0.06  0.03  0.06  0.05  0.04  0.05 |

*Normal ratio 0.7-1.3.

**The data was only collected from male controls.

***A duplicated probe signal showed in one of the 50 cases.
